# Integrative Genomic Analysis Reveals Pseudouridine Modification–Related Gene Signatures Associated With Prognosis and Therapeutic Response Patterns in Hepatocellular Carcinoma

**DOI:** 10.1155/ijog/9654215

**Published:** 2026-06-22

**Authors:** Zeying Gan, Tingyuan Fan, Wenjuan Wang, Xiao Qiao, Mingming Zhao

**Affiliations:** ^1^ Department of Critical Care Medicine, The Fourth Hospital of Changsha (Integrated Traditional Chinese and Western Medicine Hospital of Changsha, Changsha Hospital of Hunan Normal University), Changsha, China; ^2^ Department of Laboratory Medicine, The Yangzhou Clinical Medical College of Xuzhou Medical University, Yangzhou, China; ^3^ Department of Gastroenterology, Guangxi Hospital Division of The First Affiliated Hospital, Sun Yat-sen University, Nanning, China, sysu.edu.cn; ^4^ Department of Gastroenterology, The Second People′s Hospital of Huai′an, The Affiliated Huai′an Hospital of Xuzhou Medical University, Huai′an, China

**Keywords:** gene signature, genomic heterogeneity, hepatocellular carcinoma, in vitro validation, machine learning, prognosis, pseudouridine modification

## Abstract

**Background:**

Hepatocellular carcinoma (HCC) is a genomically heterogeneous malignancy with substantial variability in prognosis and therapeutic response. Pseudouridine (*Ψ*) modification is an evolutionarily conserved RNA modification involved in RNA structure stabilization and translational regulation; however, the genomic and functional relevance of pseudouridine modification–related genes (PDGs) in HCC remains poorly defined.

**Methods:**

Transcriptomic datasets and matched clinical information were collected from TCGA, GEO, and ICGC. Using feature‐selection procedures and survival modeling, we established a pseudouridine‐related risk signature. We next compared risk groups for genomic change, pathways, immune traits, and predicted drug response. Selected genes were tested in HCC cells by qPCR and CCK‐8.

**Results:**

The PDGs signature split HCC patients into high‐ and low‐risk groups with different survival and remained prognostic after adjustment. The groups also differed in mutation, pathways, immunity, and chemotherapy response. DKC1 and PUS1 knockdown reduced proliferation.

**Conclusions:**

This study identifies a PDGs signature linked with prognostic heterogeneity in HCC, supported by computational and experimental evidence.

## 1. Introduction

Hepatocellular carcinoma is the main liver cancer and a global cancer burden. It is seen as the third cause of cancer death globally [1, 2].Yearly, liver cancer has about 850,000 new cases and 600,000 deaths, over half in China, with incidence still rising [3–5]. The treatment choices for HCC include several modalities, such as surgical treatments like hepatic resection and liver transplantation; locoregional therapies including ablation, chemoembolization, and intra‐arterial treatment; systemic drugs such as sorafenib, lenvatinib, and regorafenib; also immunotherapy, radiotherapy, and yttrium‐90 radioembolization [6–9, 3, 10–12]. It should be noted that the combination of the PD‐L1 inhibitor atezolizumab and the anti‐angiogenic antibody bevacizumab has shown better overall survival (OS) and progression‐free survival in patients with unresectable or metastatic HCC when used as the first‐line regimen [13]. Even with these progress, HCC is still often diagnosed at intermediate or advanced stages due to its hidden onset, and the 5‐year survival rate remains less than 15% [14]. Traditional prognostic indicators, including alpha‐fetoprotein (AFP), AFP‐L3, and TNM stage, are still serving as the basis for present treatment decision systems, but patients who are classified into the same clinical stage often show quite different disease progression and survival results [15–20], which suggests that stage‐based prognostic evaluation has an important limitation when it is applied to individual patients. This variability clearly shows urgent need for clearer molecular predictors to improve risk grouping and personalized treatment. Posttranscriptional pseudouridylation is a common RNA regulatory process in noncoding RNAs and mRNAs [21–24]. *Ψ*, the C5‐glycosidic uridine isomer, has the only known nucleic acid C‐C glycosidic bond and, by PUS action, can affect RNA stability, interactions, turnover, and half‐life [25–30]. Twelve human PUS genes have been identified, and abnormal pseudouridylation has been associated with poor prognosis in several cancers [31–34]. In HCC, increased pseudouridine levels in serum and urine have been investigated as diagnostic and monitoring biomarkers and as a possible therapeutic target; however, the prognostic and mechanistic roles of individual PUS family members remain unclear.

Pseudouridine modification represents a structurally and functionally distinct RNA modification axis compared with other epitranscriptomic regulators such as m^6^A, with unique implications for ribosome biogenesis, translational control, and RNA decay. In contrast to studies that aggregate heterogeneous RNA modification regulators into composite signatures, a focused characterization of PDGs may reveal modification‐specific biological programs relevant to tumor biology. Nevertheless, the precise contributions of PDGs to immunotherapy response, tumor immune microenvironment (TIME) remodeling, and drug sensitivity in HCC have yet to be systematically investigated.

We combined multiomics data with machine learning–assisted prognostic analysis to develop and validate a pseudouridine‐related gene signature for HCC. We further characterized associations between PDGs‐defined risk stratification and genomic alterations, functional pathway activity, immune cell infiltration, immune checkpoint expression, and in silico chemotherapy sensitivity. Finally, loss‐of‐function experiments in HCC cell lines were performed to evaluate the functional relevance of key signature genes, providing a translational foundation for the proposed framework.

## 2. Methods

### 2.1. Raw Data Acquisition

mRNA expression data, somatic mutation profiles, and corresponding clinicopathological information for the TCGA‐LIHC cohort were downloaded from UCSC Xena. (https://xena.ucsc.edu/). The TCGA‐LIHC dataset comprised 374 hepatocellular carcinoma samples together with 50 paired nontumor liver tissues. For external validation, we also used GSE14520 from GEO (139 HCC cases) and the ICGC cohort (231 samples). Because these cohorts were different in patient features and sequencing methods, they provided independent data for testing the PDGs‐based signature.

### 2.2. Nomogram Construction

We used univariate and multivariate Cox models to test whether the calculated risk score independently predicted prognosis in the training, testing, and TCGA sets. Subgroup analyses by clinicopathological variables were also done. A nomogram for 1‐, 2‐, and 3‐year survival was built in R, and calibration curves compared predicted survival with actual outcomes.

### 2.3. Somatic Mutation Landscape Analysis

Waterfall plots showing mutation frequencies in the high‐ and low‐risk groups were produced by the “oncoplot” function in the “maftools” R package, which made the mutation features between different risk strata visually compared. The relationships between risk scores and tumor mutational burden (TMB) levels were additionally examined by using “ggplot2” visualizations.

### 2.4. Characterization of the TIME

To characterize the immune contexture of the tumors, we performed gene set variation analysis (GSVA). ssGSEA was applied to estimate the relative abundance of diverse immune cell populations and immune‐associated functional signatures. Immune infiltration was further assessed using data from TIMER 2.0, a platform integrating several deconvolution algorithms such as TIMER, CIBERSORT, quanTIseq, MCP‐counter, xCell, and EPIC. We also compared the expression of immune checkpoint‐related genes between the risk groups using the ggpubr package in R.

### 2.5. Analysis of Drug Response Sensitivity

Predicted sensitivity to nine chemotherapeutic or targeted agents was evaluated by estimating IC50 values with the pRRophetic package, and these values were then compared between the two risk groups. [35]. The resulting differences were then presented graphically with ggplot2.

### 2.6. Cell Culture

Human hepatocellular carcinoma cell lines HepG2 (RRID: CVCL_0027) and Huh7 (RRID: CVCL_0336) were purchased from ATCC. Cells were cultured in DMEM supplemented with 10% FBS, 100‐U/mL penicillin, and 100‐*μ*g/mL streptomycin at 37°C in 5% CO_2_ with saturated humidity. Routine testing confirmed that all cultured cells were free of mycoplasma contamination. Only cells in the logarithmic growth phase and with passage numbers below 20 were used for experiments.

### 2.7. siRNA‐Mediated Transfection

siRNAs targeting DKC1 and PUS1, together with a negative control siRNA (siNC), were purchased commercially. Cells were transfected at 50%–60% confluence using Lipofectamine RNAiMAX according to the manufacturer′s instructions. siRNAs were diluted in Opti‐MEM, mixed with Lipofectamine RNAiMAX, and added to cells at a final concentration of 50 nM. After 6 h, the medium was replaced with fresh complete medium. Cells were collected 24–48 h later for quantitative real‐time PCR (qPCR) or functional assays.

### 2.8. qPCR

RNA was took out by TRIzol, and amount/purity was checked by spectrophotometer. cDNA was made from 1‐*μ*g RNA with Takara kit. qPCR used SYBR Green in a real‐time system: 95°C for 30 s, then 40 cycles of 95°C for 5 s and 60°C for 30 s. GAPDH was the internal control, and expression was counted by 2^−**Δ** *Δ*Ct^. Assays were run in triplicate with three biological repeats. Primer sequences were GAPDH‐F, TTTTGCGTCGCCAGCC; GAPDH‐R, ATGGAATTTGCCATGGGTGGA; DKC1‐F, CCACAGCGGTCATCTCTACC; DKC1‐R, TTGGCAGACTCACTGTAGTCAA; PUS1‐F, ACGGATCAACTCCAACGTCC; PUS1‐R, GACTCCTGAGGAAAGCCCAC.

### 2.9. CCK‐8 Assay for Cell Viability

Cell viability was checked with CCK‐8 kit (Dojindo) by maker protocol. HepG2 and Huh7 cells were put in 96‐well plates overnight and transfected above. At 0, 24, 48, 72, and 96 h, 10‐*μ*L reagent was added into each well with 100‐*μ*L medium. After 1–2 h at 37°C, absorbance at 450 nm was measured. Growth curves were made from OD values. Experiments were triplicate and repeated at least three times.

### 2.10. Statistical Analysis

Statistics was done in R 4.1.2. Two groups used Student′s *t*‐test, whereas > 2 groups used one‐way ANOVA. Survival was estimated by Kaplan–Meier and compared by log‐rank test. Prognostic factors were screened by univariate and multivariate Cox regression. Time‐dependent ROC evaluated model prediction. *p* < 0.05 was significant.

## 3. Results

### 3.1. Analytical Screening of Pseudouridine Genes

**Figure 1:**
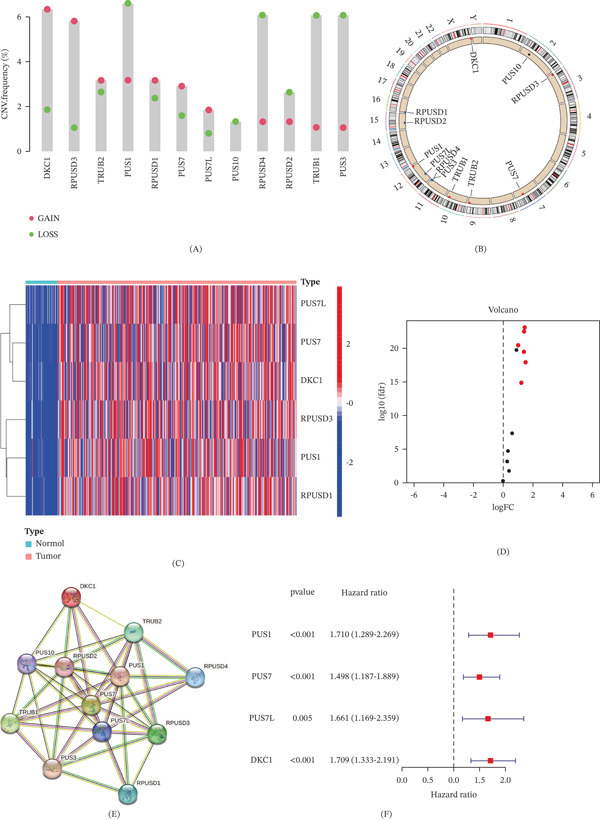
Analytical screening of pseudouridine genes in hepatocellular carcinoma (HCC). (A) Copy number variations (CNVs) of 12 pseudouridine genes (PDGs) in TCGA‐LIHC. (B) Chromosomal locations and CNV alterations of PDGs. (C, D) Heatmap and volcano plot depicting differential expression of PDGs. (E) Coexpression network of the 12 PDGs. (F) Univariate Cox regression analysis of all 12 PDGs in the complete HCC cohort.

We first analyzed copy number variation of pseudouridine genes in TCGA‐LIHC to see the genomic instability of this family. DKC1 on chromosome X and RPUSD3 on Chromosome 3 mainly showed copy number gain, whereas TRUB1 and PUS3 showed obvious loss on Chromosomes 10 and 11 (Figure 1A,B). Using the “limma” package, we found that 6 of 12 pseudouridine genes were significantly dysregulated in HCC compared with adjacent normal tissues, which was shown in the heatmap and volcano plot (Figure 1C,D). Coexpression analysis showed positive correlations among the 12 genes (Figure 1E). By univariate Cox regression, four genes with prognostic relevance were further screened out, including PUS1, PUS7, PUS7L, and DKC1 (Figure 1F).

### 3.2. Establishment and Validation of the Prognostic Model

**Figure 2:**
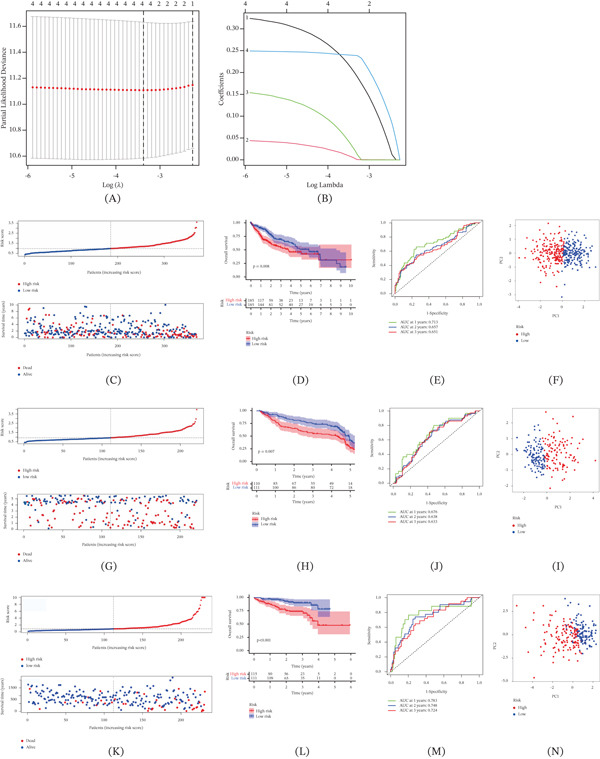
Model construction and validation across cohorts. (A) Ten‐fold cross‐validation for LASSO tuning parameter selection. (B) LASSO coefficient profiles. (C) Risk score distribution and survival status in the TCGA cohort. (D) Kaplan–Meier OS curves for high‐ versus low‐risk groups in TCGA (*p* = 0.008). (E) Time‐dependent ROC curves in TCGA. (F) PCA plot, TCGA cohort. (G–J) Corresponding analyses in the GEO cohort (KM *p* = 0.007). (K–N) Corresponding analyses in the ICGC cohort (KM *p* < 0.001).

We used LASSO Cox regression to build the PDGs model. The penalty was chosen by cross‐validation (Figure 2A,B). TCGA‐LIHC patients were scored and split by the median (Figure 2C). Low‐risk cases had longer OS (*p* = 0.008), and the 1‐, 2‐, and 3‐year AUCs were 0.713, 0.657, and 0.651 (Figure 2D,E). PCA showed the groups were not fully the same (Figure 2F). In GEO, low‐risk patients had better survival (*p* = 0.007), with AUCs of 0.676, 0.638, and 0.633 (Figure 2G–J). In ICGC, results were more clear (*p* < 0.001), with AUCs of 0.789, 0.748, and 0.724 (Figure 2K–N). So the model may keep prognostic ability.

### 3.3. Independent Prognostic Significance and Nomogram Development

**Figure 3:**
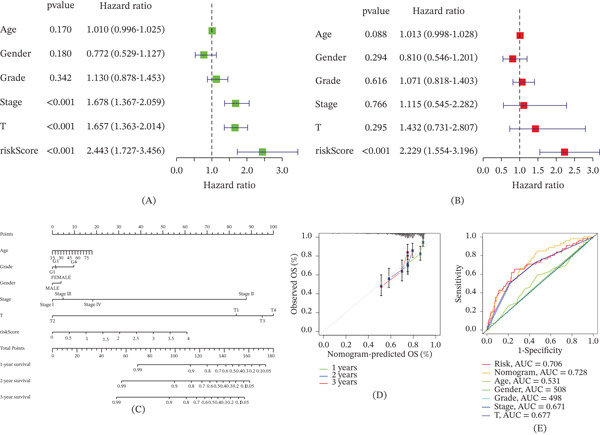
Independent prognostic value and nomogram. (A) Univariate and (B) multivariate Cox regression forest plots. (C) Nomogram for predicting 1‐, 2‐, and 3‐year OS. (D) Calibration curves. (E) Time‐dependent ROC comparison of the nomogram versus individual clinical features.

Univariate and multivariate Cox regression showed the PDGs‐derived risk score independently predicted OS after adjustment for sex, age, tumor stage, grade, and *T* stage (*p* < 0.001; Figure 3A,B). We then made a nomogram with PDGs risk score and variables for 1‐, 2‐, and 3‐year OS prediction (Figure 3C). Calibration plots looked close (Figure 3D). ROC analysis showed the PDGs risk score (AUC = 0.706) and nomogram (AUC = 0.728) were better than single variables (Figure 3E).

### 3.4. Functional Enrichment Features of Risk‐Related Genes

**Figure 4:**
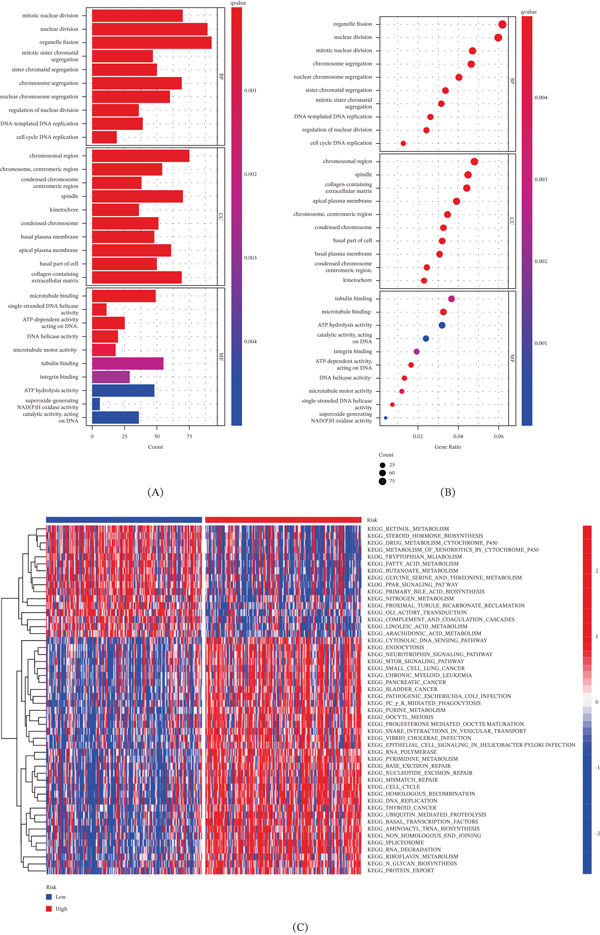
Functional enrichment analysis. (A, B) Gene Ontology (GO) enrichment results. (C) Gene set variation analysis (GSVA) comparing high‐ and low‐risk groups.

Genes from the two risk groups were analyzed by GO and KEGG. GO results suggested nuclear division, organelle fission, and chromosomal region organization (Figure 4A,B).

Further pathway‐level analysis by GSVA identified 131 pathways that differed significantly between the two risk categories (Figure 4C). Among the most representative enriched pathways were those related to RNA processing, ribosome biogenesis, cell‐cycle control, and immune‐associated signaling. These observations are in line with the recognized involvement of pseudouridine in RNA metabolism and translational regulation.

### 3.5. Analysis of TMB

TMB, defined by the frequency of somatic nonsynonymous mutations within a given genomic interval, is commonly regarded as a surrogate marker of neoantigen load and a potential predictor of immunotherapy efficacy. In this study, patients were divided into high‐ and low‐TMB groups according to the median TMB level.

**Figure 5:**
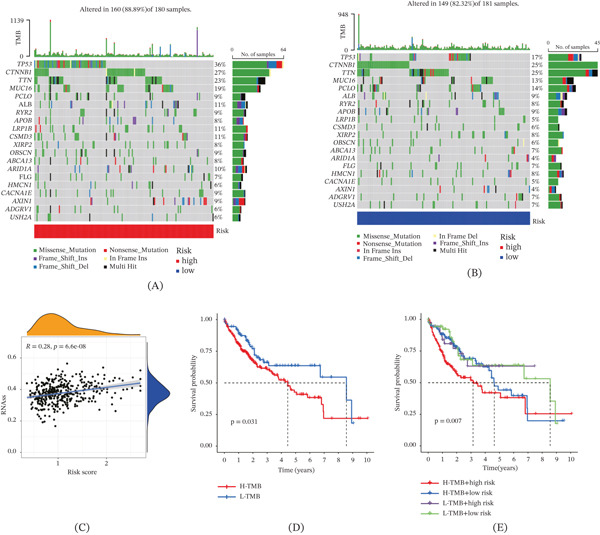
Tumor mutational burden analysis. (A, B) Mutation oncoprints for high‐ and low‐risk groups. (C) Correlation between tumor stemness index and PDGs risk score. (D) Kaplan–Meier curves stratified by TMB. (E) Combined TMB‐PDGs risk stratification survival analysis.

Oncoprint analysis showed that the mutation frequencies of several genes, including TP53, CTNNB1, TTN, MUC16, PCLO, CACNA1E, and AXIN1, differed noticeably between the high‐ and low‐risk groups (Figure 5A,B). In addition, analysis of RNA stemness scores indicated a significant association between stem cell‐like characteristics and the PDGs‐based risk score (Figure 5C). Kaplan–Meier survival analysis further revealed that patients with low TMB had a more favorable prognosis than those with high TMB (Figure 5D). When TMB status and risk score were considered together, the subgroup characterized by both low TMB and low risk exhibited the best survival outcome (Figure 5E).

### 3.6. Immune Landscape of the Tumor Microenvironment

**Figure 6:**
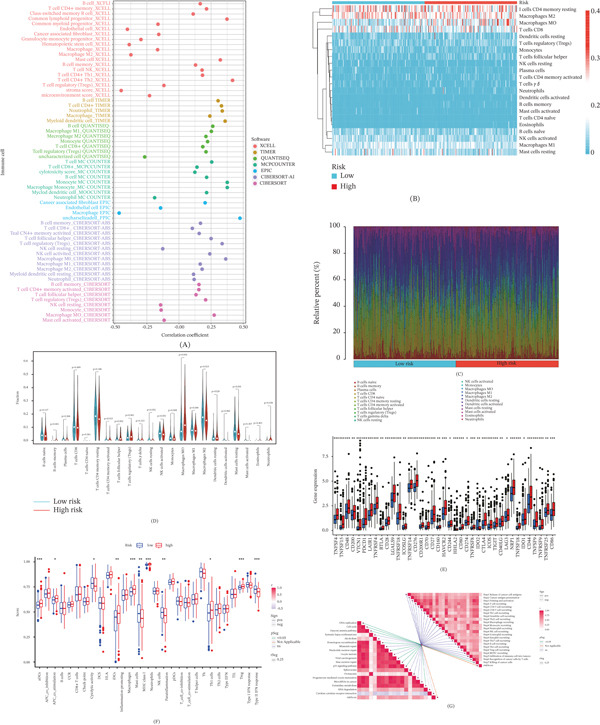
Immune microenvironment characterization. (A) Bubble plot of immune cell correlations with PDGs risk score across algorithms. (B, C) Bar plots and heatmaps of CIBERSORT immune cell fractions. (D) Comparative immune cell proportions between risk groups. (E) Immune checkpoint gene expression differences. (F) ssGSEA immune functional scores. (G) Correlations between PDGs risk score and immune pathway activity scores.

Because immune‐related pathways were enriched in the PDGs signature, we then explored the TIME in the two risk groups. Spearman correlation analysis using several immune infiltration methods showed that CD4+ and CD8+ T cells were positively associated with the PDGs risk score, whereas resting NK cells were negatively associated (Figure 6A).

Using CIBERSORT, we further estimated immune cell composition in 370 TCGA‐LIHC samples with *p* < 0.05, including 185 low‐risk and 185 high‐risk cases. The immune cell distributions were presented by bar plots and heatmaps (Figure 6B,C). Overall, immune infiltration was generally higher in the low‐risk group than in the high‐risk group, although follicular helper T cells, Treg cells, M0 macrophages, and resting dendritic cells did not completely follow this pattern (Figure 6D). These results suggest that the PDGs‐based risk groups may reflect differences in immune cell composition rather than only a general increase or decrease in immune activity. We also examined immune checkpoint–related genes and found clear differences between the two groups in several inhibitory and stimulatory checkpoint molecules (Figure 6E). Similarly, ssGSEA showed that most immune‐related functional signatures, including cytotoxic activity, costimulatory signaling, and innate immune response, were more enriched in the low‐risk group (Figure 6F). In addition, correlation analysis between the PDGs risk score and key tumor immune cycle processes showed significant positive associations with DNA replication, cell cycle progression, the Fanconi anemia pathway, homologous recombination, and nucleotide excision repair (Figure 6G). These findings indicate that PDGs‐defined subgroups have distinct immune microenvironment features, which may be associated with different responses to immunotherapy.

### 3.7. Drug Sensitivity Prediction

**Figure 7:**
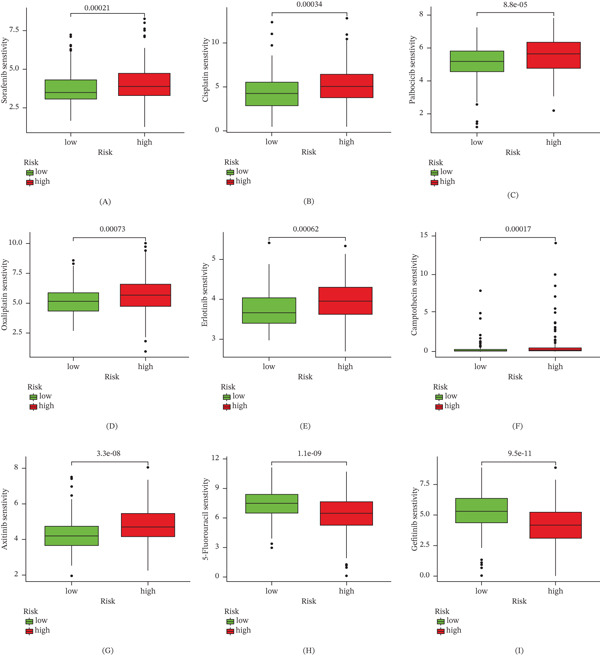
Drug sensitivity prediction. Predicted IC50 distributions for (A) sorafenib, (B) cisplatin, (C) palbociclib, (D) oxaliplatin, (E) erlotinib, (F) camptothecin, (G) axitinib, (H) 5‐fluorouracil, and (I) gefitinib.

In silico drug sensitivity analysis based on predicted IC50 values was performed to characterize therapeutic response heterogeneity across risk groups. Low‐risk patients exhibited significantly lower predicted IC50 values for sorafenib (*p* = 0.00021), cisplatin (*p* = 0.00034), palbociclib (*p* = 8.8 × 10^−5^), oxaliplatin (*p* = 0.00073), erlotinib (*p* = 0.00062), camptothecin (*p* = 0.00017), and axitinib (*p* = 3.3 × 10^−8^), indicating greater predicted sensitivity to these agents relative to the high‐risk group (Figure 7A–G). By contrast, 5‐fluorouracil and gefitinib showed higher IC50 values in the low‐risk group (Figure 7H,I), meaning a relatively weaker response. So, the PDGs risk pattern seemed to accompany different pharmacologic behaviors.

### 3.8. Validation of DKC1 and PUS1 Expression

**Figure 8:**
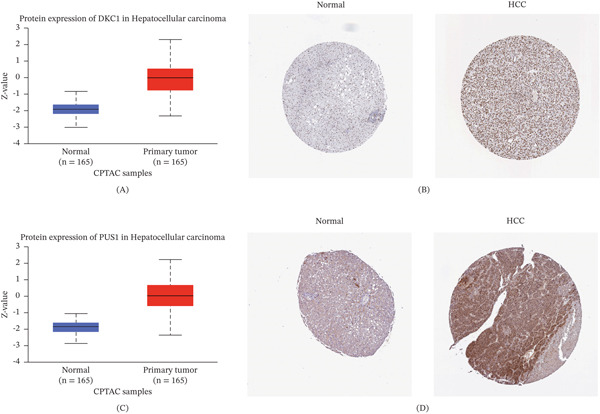
Validation of DKC1 and PUS1 expression. (A, B) Protein expression levels of DKC1 and PUS1 in HCC versus normal tissues from Ualcan and HPA databases. (C, D) Immunohistochemical validation of DKC1 and PUS1 expression.

Protein expression of DKC1 and PUS1 was checked in Ualcan and HPA. Both proteins were higher in HCC tissue than in adjacent normal liver (Figure 8). Immunohistochemistry was generally consistent with the mRNA overexpression, which further supports their relevance in the PDGs framework.

### 3.9. Functional Validation of DKC1 and PUS1 in HCC Cells

**Figure 9:**
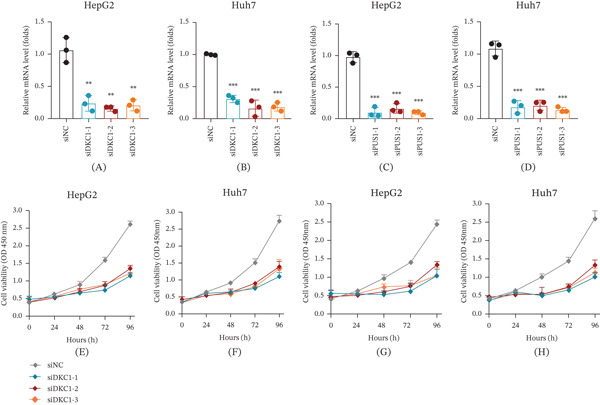
Functional validation of DKC1 and PUS1 in HCC cells. (A, B) qPCR quantification of DKC1 mRNA knockdown in (A) HepG2 and (B) Huh7 cells with three independent siRNAs versus siNC. (C, D) PUS1 mRNA knockdown in (C) HepG2 and (D) Huh7 cells. (E, F) CCK‐8 proliferation curves following DKC1 knockdown. (G, H) CCK‐8 proliferation curves following PUS1 knockdown. Expression normalized to GAPDH; fold changes relative to siNC. Data: mean ± SD, ≥ 3 independent experiments.  ^∗∗^ *p* < 0.01,  ^∗∗∗^ *p* < 0.001.

To verify the role of key PDGs genes, loss‐of‐function assays for DKC1 and PUS1 were carried out in HCC cells. qPCR showed that three DKC1 siRNAs effectively reduced DKC1 expression in HepG2 and Huh7 cells compared with siNC (Figure 9A,B). Three PUS1 siRNAs also produced stable knockdown in both cell lines (Figure 9C,D).

CCK‐8 assays showed that DKC1 knockdown clearly reduced the growth of HepG2 and Huh7 cells over 96 h compared with siNC controls (Figure 9E,F). PUS1 silencing showed a similar inhibitory effect (Figure 9G,H). Since all three siRNAs produced similar results, the possibility of off‐target effects may be relatively low. These findings suggest that DKC1 and PUS1 are important for maintaining HCC cell proliferation and support their inclusion in the PDGs prognostic model.

## 4. Discussion

Hepatocellular carcinoma is a global health problem, with high incidence, high mortality, and heterogeneity even among tumors with the same histopathology diagnosis [36, 37]. This heterogeneity comes from genomic, epigenetic, and microenvironmental factors, and these differences are now considered meaningful, not only descriptive [38]. Conventional staging systems are still important in clinic, but they explain only part of disease behavior because they mainly depend on tumor burden and liver function, whereas molecular programs driving progression, recurrence, and treatment response are not reflected [39]. Therefore, adding molecular features into risk assessment is important because HCC prognosis should be judged not only by stage but also by tumor biology and tumor–host interaction [40]. In this study, pseudouridine modification–related genes were identified as a relevant axis, and pseudouridine‐associated transcriptional states were linked with survival, mutation pattern, immune context, and predicted therapy response.

The significance of this work is related to the distinct role of pseudouridylation in epitranscriptomic regulation. RNA modifications are increasingly recognized as regulators of cancer phenotype through affecting RNA stability, translation, splicing, and stress adaptation [41–43]. Pseudouridine is not simply another RNA mark, but a structural modification that may alter RNA folding, ribosome function, and decoding fidelity, therefore influencing posttranscriptional processes [44]. Previous studies in dyskerin‐deficient systems showed impaired pseudouridylation can disrupt ribosomal ligand binding and selectively alter translation of transcripts involved in cell‐cycle control and tumor suppression, including p27‐ and p53‐related pathways [45–48]. These findings suggest pseudouridine dysregulation is maybe more than a correlative feature in cancer. Earlier studies also examined pseudouridine as a biomarker in several malignancies, including liver cancer [41, 49–55]. However, pseudouridine‐related genes have not been systematically evaluated as one integrated prognostic framework in HCC.

We established a four‐gene PDGs signature including PUS1, PUS7, PUS7L, and DKC1, which stratified patients into distinct risk groups across TCGA and two external cohorts. The risk score remained independently associated with OS after adjustment of clinicopathological variables, and the nomogram improved predictive performance compared with clinical features. The signature was also associated with pathway activity, mutational burden, stemness, immune state, and predicted drug sensitivity. These findings suggest the PDGs signature captures a meaningful biological aspect of HCC. This plausibility is also supported by component gene functions: PUS1 has been linked with aggressive cancer behavior [56], whereas DKC1, as catalytic core of the H/ACA ribonucleoprotein complex, participates in pseudouridylation, telomere maintenance, translational control, mitotic integrity, stemness‐related programs, and miRNA‐related regulation [57–60].These functions are of direct relevance to HCC, a disease in which proliferative drive, translational plasticity, and replicative stress are central features of aggressive tumors. The association of DKC1 overexpression with poor outcome in other malignancies and its functional contribution to tumor growth in renal and lung cancer models further support its candidacy as a driver rather than a bystander [61, 62]. Within this broader context, the recurrence of PUS1‐linked biology across cancer datasets remains notable [56].

Our experimental observations provide an additional layer of support. Although the present study was not designed as a mechanistic dissection of each PDGs component, silencing of DKC1 and PUS1 consistently impaired proliferation in HepG2 and Huh7 cells. This is an important point. Multiomics prognostic signatures are increasingly easy to construct computationally, but many remain difficult to interpret biologically because the genes involved are never functionally tested. By showing that downregulation of two central components of the signature attenuates tumor cell growth, we establish at least a first experimental bridge between the prognostic model and malignant phenotype. At the same time, these results should be interpreted with appropriate caution. The growth effects observed in vitro do not specify whether these genes act through global translational control, altered ribosome biogenesis, stress response pathways, checkpoint regulation, or additional context‐dependent mechanisms. Nor do they exclude the possibility that the prognostic capacity of the full signature reflects both cell‐autonomous and microenvironmental effects. Still, when survival model, pathway enrichment, and loss‐function data are looked at together, PDGs seem not only passive signs of proliferation. They may also help arrange a malignant condition where RNA modification machinery supports HCC needs.

A notable part of our findings is the relation between PDGs‐defined risk and genomic instability. The high‐risk group showed a different mutation pattern, including genes like TP53, CTNNB1, and AXIN1, with links to stemness‐like features. Although our analysis was not made to prove causal order, these observations suggest pseudouridine‐related states may coexist with, or may help maintain, a more permissive tumor phenotype. Enrichment of nuclear division, organelle fission, and chromosomal region organization fits a proliferative program under replicative pressure. High‐risk tumors may also depend on stronger genome maintenance to bear stress. Clinically, PDGs may mark a broader aggressive state. The low TMB plus low PDGs‐risk subgroup had the best survival, suggesting mutational load alone does not explain outcome as well as transcriptional state does.

This point is worth emphasizing because TMB is frequently interpreted through a simplified immuno‐oncology lens in which higher mutational load is expected to improve neoantigenicity and immunotherapy responsiveness. In HCC, however, the relationship between TMB, immune activation and clinical benefit is more complex. The liver is a uniquely tolerogenic organ, and mutational burden may coexist with immune exclusion, stromal suppression or dysfunctional inflammatory states that do not translate into productive antitumor immunity. In our study, higher TMB was associated with worse survival, and the prognostic effect of PDGs risk remained evident when considered jointly with TMB. These results suggest that pseudouridine‐related transcriptional programs may contextualize genomic complexity by distinguishing tumors that are not merely mutated, but biologically organized around distinct proliferative and immune ecologies. Such a framework may ultimately prove more useful than any single biomarker alone, particularly in a disease as molecularly and clinically heterogeneous as HCC.

The immune analyses further support the idea that PDGs stratification captures biologically divergent disease states. The low‐risk group appeared more immune‐rich, whereas the high‐risk group looked more linked with proliferation and DNA repair. In HCC, immune context is not only whether lymphocytes are present; balance among effector cells, suppressive myeloid cells, stromal structure, and immune‐cell function also matters [63, 64]. Our data suggest PDGs‐defined tumors are maybe at different points on this axis, with stronger cytotoxic activity, costimulatory signaling, and innate immune response in the low‐risk phenotype.

Previous HCC studies reported that follicular helper T cells can become functionally impaired with PD‐L1 signaling, causing lower cytokine production and weaker B‐cell help [65, 66]. This fits our results and suggests high‐risk tumors may stay more dysfunctional even when some immune components are still detectable. Altered mast‐cell and macrophage‐subset abundance may also matter, because hepatic immune‐landscape deviations in HCC can affect antigen presentation, tissue remodeling, and local inflammatory tone [67]. In our cohort, lower abundance of some immune populations in the high‐risk state, together with altered checkpoint expression, may reflect compromised immune surveillance.

This is especially relevant in advanced HCC, where liver immune tolerance, cirrhosis, and ongoing inflammatory injury make antitumor immunity more complicated and reduce therapeutic durability [68, 69]. Immunotherapy changed the treatment landscape, but benefit is still heterogeneous, and single‐agent checkpoint blockade usually gives only modest and transient responses in unselected populations [70, 71]. Therefore, biomarkers for better patient selection remain urgent, and our results suggest the PDGs risk score may help identify tumors with different checkpoint landscapes and immune states.

CTLA‐4 regulates early T‐cell priming by competing with CD28 for B7 ligands and weakening co‐stimulatory signaling [72]. Clinical experience with tremelimumab and other checkpoint‐directed therapies in HCC showed that immune modulation can produce disease control in selected patients, although responses are variable and not always complete [73].Our observation that PDGs‐defined groups differ in several inhibitory and stimulatory checkpoint molecules suggests pseudouridine‐related biology may intersect with immune escape circuitry at a systems level. It also agrees with current HCC treatment paradigms, which increasingly rely on combination strategies to overcome non‐redundant mechanisms of immune resistance [74, 75]. If validated in immunotherapy‐treated cohorts, a PDGs‐based framework might help identify patients more likely to benefit from combinational approaches rather than monotherapy.

Our drug‐sensitivity analyses extend the clinical relevance of the model beyond prognosis. Low‐risk tumors were predicted to be more sensitive to several agents, including sorafenib, cisplatin, oxaliplatin, erlotinib, camptothecin, axitinib, and palbociclib, whereas sensitivity to 5‐fluorouracil and gefitinib showed the opposite pattern. Although these estimates are computational rather than experimental, the pattern is nonetheless informative. It suggests that PDGs‐defined states may capture pharmacologic liabilities linked to the broader transcriptional architecture of the tumor. One plausible interpretation is that the immune‐enriched, less genomically stressed low‐risk phenotype retains vulnerabilities to several targeted and cytotoxic agents, whereas the high‐risk phenotype may represent a more therapy‐adaptive state with enhanced capacity to withstand proliferative or genotoxic challenge. Equally, the association between high‐risk tumors and cell‐cycle or DNA repair pathways raises the possibility that such tumors may depend on compensatory stress‐tolerance circuits not directly interrogated here. This does not yet justify therapeutic claims, but it supports the idea that pseudouridine‐related stratification may serve as a practical scaffold for future drug‐response modeling. Translationally, a biomarker suggesting treatment weak points is more useful than one that only predicts survival.

More broadly, our results support that RNA regulation in HCC is not just secondary to genomic change but also helps tumor fitness. Pseudouridylation seems important mostly because it is linking ribosome work, RNA steadiness, and translation choosing in a way that is probably not random. Tumors with bigger biosynthetic needs may rely on this machinery more strongly, so the four‐gene signature may indicate a deeper biological needing instead of being only a descriptive classifier. That could partly account for the stable signal across datasets. The presence of PUS7 and PUS7L together with PUS1 and DKC1 suggests a kind of coordinated pseudouridylation‐related condition. Future work should more directly check pseudouridine deposition, identify targets, and examine how this is connecting with oncogenic signaling and immune remodeling. Still, the present data gives a starting basis. This study also has several strengths, including outside cohort validation, integrated multi‐omic and drug‐response analyses, and experimental support for DKC1 and PUS1 in two HCC cell lines, which together makes the PDGs signature more believable. But limitations also exist: The study is retrospective, immune results come from computational deconvolution, drug sensitivity is only in silico, and the mechanistic roles of DKC1, PUS1, PUS7, and PUS7L are still not being fully clarified. We did not directly measure pseudouridine abundance or transcript‐specific pseudouridylation in tumor tissue, which means that the biological state inferred by the signature remains indirect. The TCGA‐centric discovery framework may not fully capture the etiologic and ethnic diversity of global HCC, including populations in which viral hepatitis, aflatoxin exposure or metabolic liver disease contribute differently to tumor evolution and treatment response. Prospective validation in more diverse cohorts, especially those with modern immunotherapy and targeted‐therapy annotation, will be essential.

This study identifies pseudouridine modification–related genes as a clinically and biologically informative framework in HCC. The four‐gene PDGs signature stratified survival across multiple cohorts, remained independent of standard clinicopathological factors, and corresponded to distinct proliferative, genomic, immune, and pharmacologic states. Our DKC1/PUS1 knockdown kind of proves this pathway helps tumors keep going. More broadly, pseudouridine biology may be an overlooked HCC layer, relevant for risk judging, mechanism study, and therapy sorting. Since useful biomarkers stay limited, PDGs may be a workable start for seeing how RNA modification shapes liver‐cancer behavior.

## 5. Conclusion

This study identified a pseudouridine modification–related gene signature by machine learning that could independently stratify HCC prognosis across several cohorts. Integrated analyses suggested that PDGs risk groups had different mutation patterns, pathway activities, and immune microenvironment features. In vitro experiments also supported that DKC1 and PUS1 are important for maintaining HCC cell proliferation. Together, these findings provide a framework linking pseudouridine biology with prognostic heterogeneity in HCC and may support future mechanistic and translational studies.

## Author Contributions

The study was conceived and designed by X.Q. and M.Z. Z.G., T.F., and W.W. were responsible for preparing the initial manuscript, conducting the literature survey, and collecting the relevant data. These three authors also completed the data processing, statistical analysis, and figure preparation, as well as the in vitro experimental work. X.Q. and M.Z. participated in revising and refining the final version of the manuscript. Z.G., T.F., and W.W. have contributed equally to this work.

## Funding

This study was supported by the Jiangsu Health International Exchange Program.

## Disclosure

All authors read and approved the submitted manuscript. The authors take full responsibility for the final content of the manuscript.

## Ethics Statement

The authors have nothing to report.

## Conflicts of Interest

The authors declare no conflicts of interest.

## Data Availability

The data used and evaluated in this research can be obtained from publicly accessible databases, including The Cancer Genome Atlas (TCGA), the Gene Expression Omnibus (GEO), and the International Cancer Genome Consortium (ICGC) through their respective official platforms.
